# Speciation of Candida using CHROMagar in cases with oral epithelial dysplasia and squamous cell carcinoma

**DOI:** 10.4317/jced.54737

**Published:** 2018-07-01

**Authors:** Supriya Hulimane, Ramya Maluvadi-Krishnappa, Shaila Mulki, Harishchandra Rai, Anitha Dayakar, Meghashree Kabbinahalli

**Affiliations:** 1 Senior lecturer, Department of Oral Pathology and Microbiology, KVG Dental College and Hospital, Sullia - 574 237, D.K, Karnataka, India

## Abstract

**Background:**

*Candida albicans* is most frequently isolated from oral cavity but identification of other *Candida* species such as *C. tropicalis, C. krusei, C. glabrata* & *C. dubliniensis* is increasing proportionately. A constant rise in immuno-suppressed patients, widening range of recognized pathogens, and resistance to antifungal drugs are contributing factors which stress the need for species identification of *Candida*, an opportunistic pathogen. Objectives: 1. To detect the prevalence of *Candida albicans* and Non albicans *Candida albicans* (NAC) species in the oral cavity of patients with epithelial dysplasia, Oral squamous cell carcinoma (OSCC) and healthy controls. 2. To identify and differentiate Candidal species using CHROMagar, a differential media.

**Material and Methods:**

The study included smears from 50 patients with histopathological confirmation of epithelial dysplasia & OSCC and 50 normal controls. *Candida albicans* was identified using Sabouraud dextrose agar media (SDA) as primary culture followed by species identification using CHROMagar on the basis of colony color and morphology.

**Results:**

*Non albicans candida* predominated (66%) over *Candida albicans* (34%) in speciation on CHROMagar media in the study group. Non albicans Candida species isolated were *C. tropicalis* (38%), *C. glabrata* (24%) and 2 cases showing polyfungal population of *C. albicans* & *C. glabrata*.

**Conclusions:**

Species level isolation of *Candida* helps in early identification of resistant non *Candida* strains and prompt treatment of the cases there by preventing the dissemination of infection in case of immuno-compromised individuals. The data presented also supports the use of CHROMagar *Candida* as a pertinent media for the rapid identification of *Candida* species directly from clinical specimens in resource challenged settings, which could be helpful in developing appropriate therapeutic strategy and management of patients.

** Key words:**Candida, CHROMagar, epithelial dysplasia, oral cancer.

## Introduction

Oral squamous cell carcinoma, the most common form of oral cancer, represents up to 80–90 % of all malignancies of the oral cavity. Though risk factors like smoking, alcohol, ultraviolet radiation and poor oral hygiene play an important role in the pathogenesis, occurrence of OSCC has also been associated with *Candida* infections, although the underlying pathogenic mechanisms are poorly understood (1).

Genus *Candida* is responsible for a variety of infections in humans ranging from surface infections to systemic candidiasis (2). Though a part of normal microbial flora, any alteration in immune status will promote the proliferation of endogenous *Candida*. *Candida spp.* have several attributes that may promote oral cancer development, such as the ability to produce carcinogens (e.g. nitrosamines), metabolize procarcinogens or induce inflammation (3). Although Candida albicans is the most predominant species isolated, other candidal species identified include *Candida glabrata, Candida dubliniensis, Candida guilliermondii, Candida krusei, Candida lusitaniae, Candida parapsilosis, Candida tropicalis and Candida kefyr* (4).

Conventional methods of yeast identification like assimilation and fermentation characteristics are reported to be inconvenient and beyond the expertise range available in local laboratories (5). Chromogenic media available for rapid identification of yeast yield microbial colonies with varying pigmentation of secondary substrates that react with enzymes secreted by micro-organisms. These media are species-specific, allowing the organisms to be identified to the species level by their color and colonial characteristics. It is easy to perform, requires less time and is cost effective too (6).

The present study was aimed to study the frequency oral *Candida* and NAC in individuals with epithelial dysplasia & OSCC and their species level identification. Identification of the *Candida* isolates were done by cultivation in SDA & Gram staining followed by differentiation of species based on colony morphology in CHROMagar.

## Material and Methods

The study design was approved by the Institutional Research Ethics committee. Fifty histopathologically diagnosed cases of oral epithelial dysplasia or OSCC visiting the institution were involved in the study along with 50 normal controls, after obtaining their written informed consent. Personal and clinical details were obtained from all the patients. Patients clinically suspected with oral candidiasis, currently receiving treatment with antibiotics, antifungals or steroids or who had received in the previous 3 months were excluded from the study.

**Figure 1:**
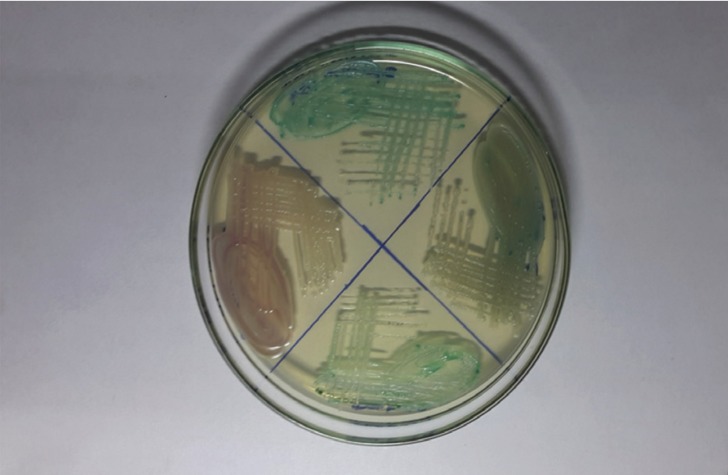
CHROMagar culture plate showing colonies of *C.albicans* & *C.glabrata*.

Smears were obtained from lesional sites in the study group and buccal mucosa in normal controls by swabbing lesions in the oral cavity with a sterile cotton swab. These swabs were then inoculated onto SDA and incubated at 37°C for 48 hours. Gram staining was done to identify *Candida* species. Yeast colonies growing on each SDA tube were resuspended and 10 µL of suspension solution was used to inoculate plates with CHROMagar medium. Inoculated plates were incubated at 37°C and read for up to 7 days. Plates were observed for fungal growth using morphology and colour to determine the presence of yeasts. *C.albicans* by growth was presumptively identified as light to medium green colonies, *C.tropicalis* as steel blue colonies accompanied by purple pigmentation diffused into surrounding agar, and *C. glabrata* by growth as large, fuzzy, rose colored colonies with white edges, after incubation for 48 hours at 37 °C, as given in manufacturer guidelines and also reported in several studies (1,4) (Fig. 1). Isolates that produced dark-green colonies were suspiciously considered for *C. dubliniensis*, while light-green colonies were presumptively identified as *C. albicans* (5) and further confirmed by germ tube test on pooled serum at 2 hrs as *C.albicans*.

Statistical work: Descriptive statistical analysis was carried out using SPSS 20 software version. Chi square test was used to find the significance & a *p* value < 0.001was considered as significant.

## Results

Of the hundred subjects, *Candida* species were isolated from all the 50 cases and 2 of the 50 normal controls. 32 cases of epithelial dysplasia and 18 cases of OSCC in histopathologically varying grades in the age group of 35-65 years showed the presence of *C. albicans* and NAC. High level of *Candida* colonization was seen in study group (N=50,100%) when compared with the colonization in normal controls (N=2,4%).

Chi –square analysis of colonisation of C. albicans and NAC among the study groups were found to be statistically significant ( Table 1).

**Table 1:**
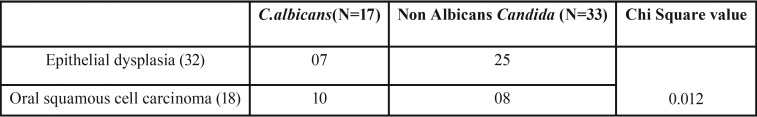
Association of *C.albicans* & Non Albicans *Candida* within the study group.

When association of *C. albicans* & NAC was compared with different histopathological grades of epithelial dysplasia and squamous cell carcinoma by using chi square analysis the data showed no evidence of a significant relationship ( *p*>0.001) ( Table 2).

**Table 2:**
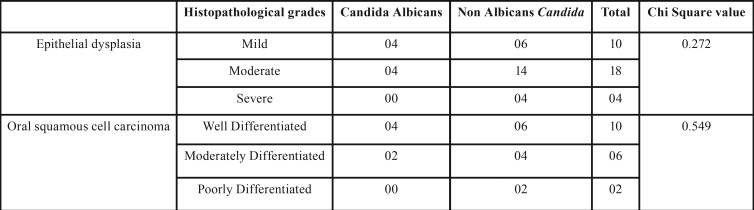
Colonization of *C. albicans* & Non Albicans *Candida* among histopathological grades of epithelial dysplasia & Oral squamous cell carcinoma.

Further species identification using CHROM agar showed prevalence of other *Candida* species like *C.Tropicalis* (N=20) & *C.Glabrata* (N=13). Two positive cultures showed polyculture of *Candida* species and the strains observed was identified as *C. albicans* and *C. glabrata* (  Table 3).

**Table 3:**
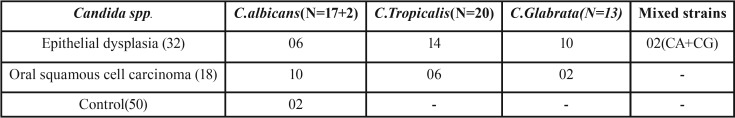
Speciation of Candida in study and control group.

*C. dubliniensis* showing phenotypic similarity to *C. albicans* was not detected in the present study.

## Discussion

Despite advances in investigation and treatment, oral cancer still ranks higher in its incidence worldwide. In the Indian subcontinent the occurrence is greatly influenced by oral habits like tobacco chewing and smoking. *C. albicans*, an oral commensal is the most important yeast pathogen in humans. Its transition to an opportunistic pathogen may be associated with the virulence of the organism and the host factors. Previous studies have shown high frequency of isolation of *Candida* species in patients with epithelial dysplasia compared to healthy controls (7,8).

Though CHROMagar *Candida* media is not as cost effective as SDA, it is found to be a satisfactory isolation media for oral cavity specimens, allowing rapid and accurate identification of yeast colonies and easy recognition of mixed cultures which favors its use in mycology laboratories (9). Previous studies have also shown CHROMagar to parallel the results of conventional method and its superiority to SDA in terms of suppressing the bacterial growth (10). Our study was able to identify *Candidal spp.* with accuracy in all samples proving sensitivity of the media and also advantage of recognition of mixed strains.

Previous studies have demonstrated that *C. albicans* is the most frequently isolated yeast species from oral cavity in orthodontic and oral medicine patients (11). Negative culture in controls in our study may indicate their absence in healthy hosts. An array of local, systemic exogenous and fungal survival factors could have raised the prevalence of *candidal* carriage and population in patients with oral lesions explaining the positivity in the study group. In yet another study to correlate the prevalence of *candidal* species in the oral cavity of OSCC patients undergoing radiation therapy and quantitative analysis of the strains using CHROMagar demonstrated a drastic increase in colonization of *C. tropicalis* and *C. glabrata* population (12) further stressing the need for identification during therapy also.

In our study, detection frequency of *Candida* species was significantly higher in subjects with oral lesions than in those with healthy oral mucosa, thus supporting an association between *Candida* species and oral lesions. This was similar to studies which employed other methods for isolation of *Candida* (13,14). Speciation using CHROMagar showed equal or a slight predominant presence of Non albicans comparatively (N=33, 66%) ( Table 1).

*Non albicans Candida* species have been described as emerging species which cause fungal infections in immunocompromised hosts (15). These NAC species lack many of the virulence factors present in the virulent C. albicans i.e. the ability to form hyphae and phenotypic switching (16). Studies on antifungal resistance have shown that Azole resistance was more in NAC spp. as compared to *C. albicans*. This is of concern because azoles like fluconazole are among the most commonly used antifungal agents for the treatment of candidiasis (17). This stresses the importance of isolating & identifying *Candida* species.

Another emerging species *C. dublinesis* is ruled out by germ tube growth at 2 hrs of all *C. albicans* samples on pooled human serum. Though not identified in the present study, nevertheless, this NAC spp. showing phenotypic similarity to *C. albicans* cannot be neglected in oral lesions.

## Conclusions

Present study demonstrated the predominance of other NAC spp. in the oral cavity of patients with lesions concluding that NAC spp. has emerged as an important cause of infections. Its isolation from clinical specimens can no longer be ignored as non-pathogenic isolate nor as a contaminant. Furthermore, use of chromogenic media was found to be beneficial for rapid isolation and direct identification of *Candida* species especially the medically important one. Further large-scale studies, are required in order to analyze the association between non-*C. albicans* species and OSCC to establish the pathogenic potentiality of non-*C. albicans* species.
